# Improved xylose tolerance and 2,3-butanediol production of *Klebsiella pneumoniae* by directed evolution of *rpoD* and the mechanisms revealed by transcriptomics

**DOI:** 10.1186/s13068-018-1312-8

**Published:** 2018-11-09

**Authors:** Xue-Wu Guo, Yu Zhang, Lu-Lu Li, Xiang-Yu Guan, Jian Guo, De-Guang Wu, Ye-Fu Chen, Dong-Guang Xiao

**Affiliations:** 1 Key Laboratory of Industrial Fermentation Microbiology of Ministry of Education, Tianjin Industrial Microbiology Key Lab, College of Biotechnology of Tianjin University of Science and Technology, Tianjin, 300547 China; 2 Tianjin Food Safety & Low Carbon Manufacturing Collaborative Innovation Center, Tianjin, 300547 China; 3 Tianjin Engineering Research Center of Microbial Metabolism and Fermentation Process Control, Tianjin, 300457 China

**Keywords:** Xylose, 2,3-Butanediol, *Klebsiella pneumoniae*, Sigma factor, *rpoD*

## Abstract

**Background:**

The biological production of 2,3-butanediol from xylose-rich raw materials from *Klebsiella pneumoniae* is a low-cost process. *RpoD*, an encoding gene of the sigma factor, is the key element in global transcription machinery engineering and has been successfully used to improve the fermentation with *Escherichia coli*. However, whether it can regulate the tolerance in *K. pneumoniae* remains unclear.

**Results:**

In this study, the kpC mutant strain was constructed by altering the expression quantity and genotype of the *rpoD* gene, and this exhibited high xylose tolerance and 2,3-butanediol production. The xylose tolerance of kpC strain was increased from 75 to 125 g/L, and the yield of 2,3-butanediol increased by 228.5% compared with the parent strain kpG, reaching 38.6 g/L at 62 h. The RNA sequencing results showed an upregulated expression level of 500 genes and downregulated expression level of 174 genes in the kpC mutant strain. The pathway analysis further showed that the differentially expressed genes were mainly related to signal transduction, membrane transport, carbohydrate metabolism, and energy metabolism. The nine most-promising genes were selected based on transcriptome sequencing, and were evaluated for their effects on xylose tolerance. The overexpression of the *tktA* encoding transketolase, *pntA* encoding NAD(P) transhydrogenase subunit alpha, and *nuoF* encoding NADH dehydrogenase subunit F conferred increased xylose consumption and increased 2,3-butanediol production to *K. pneumoniae*.

**Conclusions:**

These results suggest that the xylose tolerance and 2,3-butanediol production of *K. pneumoniae* can be greatly improved by the directed evolution of *rpoD.* By applying transcriptomic analysis, the upregulation of *tktA*, *pntA*, and *nuoF* that were coded are essential for the xylose consumption and 2,3-butanediol production. This study will provide reference for further research on improving the fermentation abilities by means of other organisms.

**Electronic supplementary material:**

The online version of this article (10.1186/s13068-018-1312-8) contains supplementary material, which is available to authorized users.

## Introduction

Renewable resources have attracted great attention due to the growing demand for energy and chemicals [1, 2]. Xylose is the most abundant sugar in lignocellulosic materials, except in glucose [3]. Therefore, its efficient utilization is crucial for the utilization of the lignocellulosic biomass, an abundant, geographically ubiquitous, and potentially cheap renewable resource, for the enhanced production of fuels and chemicals [4].

The strain with high tolerance to substrate, production, and toxic materials is quite essential for biotransformation. Some species can take advantage of xylose, such as *Bacillus polymyxa*, *Enterobacter cloacae*, *Klebsiella pneumoniae*, *Pichia stipitis*, *Candida shehatae*, and *Pachysolen tannophilus.* Microorganisms have two kinds of xylose utilization pathways, namely, the xylose reductase/xylitol dehydrogenase (XR/XDH) and xylose isomerase (XI) pathways. Xylose isomerase is present in *K. pneumoniae*, which can convert xylose into xylulose immediately through isomerization. Eventually, xylulose is metabolized through the pentose phosphate pathway (PPP) in the form of xylulose-5-phosphate [5–7]. The *K. pneumoniae* is among the microbes that can metabolize xylose naturally [8–10]. However, its xylose tolerance is unsatisfactory. In previous studies, *K. pneumoniae* has been used to produce 2,3-butanediol in glucose medium, but xylose has rarely been reported. Furthermore, *K. pneumoniae* cannot fully metabolize 2,3-butanediol by fermenting xylose at a high concentration (exceeding 70 g/L).

With the rapid development of molecular biology, genetic engineering, and other fields of biotechnology, the effects of many genes on most cellular phenotypes have been generally accepted. As a result, engineering a desired phenotype would be facilitated enormously by simultaneous multiple gene modification; however, the capacity to introduce such modification is limited [11–15]. Cellular systems have optimized the capacity to self-regulate thousands of genes by fine tuning components of global transcription machinery. Based on this theory, global transcription machinery engineering was created [16].

Sigma (*σ*) factor is an important global transcriptional regulatory factor for bacteria [17]. In a previous study, gene *rpoD* (encoding gene of the *σ* factor) has been transformed and expressed in *Escherichia coli* and other bacteria, thus promoting the growth velocity and the tolerance for ethanol or other compounds of the thallus [18, 19]. However, whether it can regulate the tolerance abilities in other strains remains unclear.

Due to the wide substrate spectrum and high adaptability of *K. pneumoniae*, it has been chosen as a producer of high-value biological and chemical products, including 2,3-butanediol and acetoin. In this study, *K. pneumoniae* (kpC strain) with a high xylose tolerance and high 2,3-butanediol yield was screened through error-prone PCR to introduce mutations to *rpoD*. Afterward, the global transcriptional regulation mechanism of the kpC strain was studied using next-generation RNA sequencing (RNA-seq) to enhance the understanding of the kpC phenotype. Finally, the regulatory mechanism of xylose tolerance was investigated by gene deletion and expression by means of the transcription results.

## Results

### Examination of the xylose tolerance of the kpG strain

**Fig. 1:**
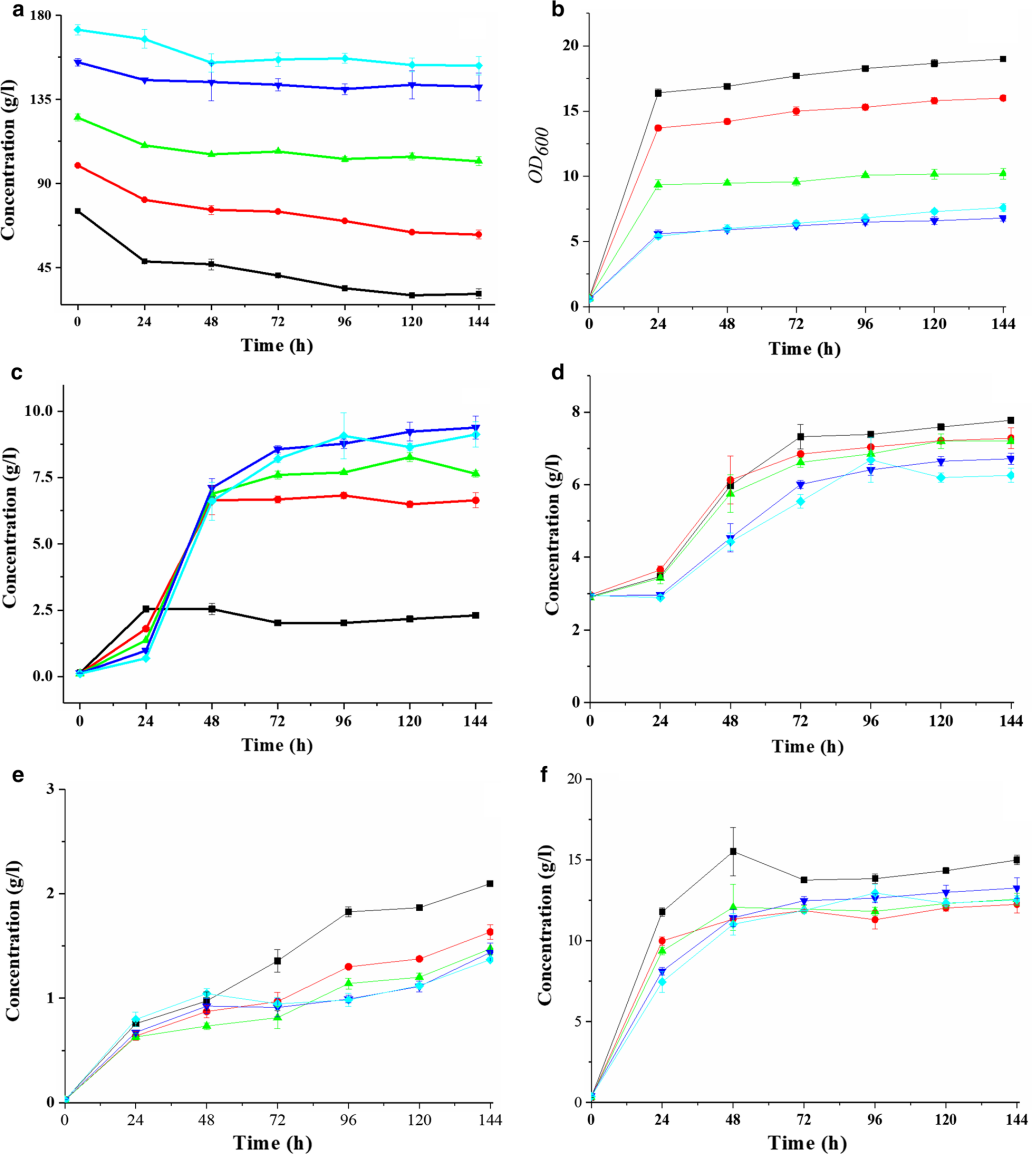
Results of fermentation with different concentrations of xylose (**a** xylose, **b** b:growth curve, **c** succinic acid, **d** acetic acid, **e** acetoin, **f** 2,3-butanediol). The initial concentrations of xylose are 75 g/L (square), 100 g/L (circle), 125 g/L (up-triangle), 150 g/L (down-triangle), and 175 g/L (diamond)

The kpG was cultured at xylose concentrations of 75 100, 125, 150, and 175 g/L in a shake flask, and the concentrations of the carbon source and metabolite were detected every 24 h. The results are listed in Fig. 1.

From Fig. 1, the highest utilization efficiency of xylose occurred at an initial xylose concentration of 75 g/L, where the logarithmic phase lasted for 72 h. As xylose concentration increased, the rate of xylose consumption decreased, and the logarithmic phase shortened. As the initial xylose concentration was increased to 125 g/L, the biomass accumulated only within the early 12 h. The results showed that for kpG, the threshold value of xylose tolerance was 75 g/L. Considering the xylose-tolerant strains, an initial xylose concentration of 125 g/L was used as a filter condition.

Furthermore, with the decreasing xylose consumption, the syntheses of acetoin, 2,3-butanediol, and acetic acid decrease. In contrast, succinic and lactic acids are amassed from 4 to 5 times and 1.5 to 4 times, respectively. The experimental results show that fermentation is limited when the concentration of xylose is excessive. Along with the changing of product, this limitation might be caused by the unbalance of NADPH/NADP^+^ and ATP/ADP, because the syntheses of succinic and lactic acids utilize NADPH and ADP, accumulating NADP^+^ and ATP in the cells [20].

### Isolation of xylose-tolerant mutant

**Fig. 2:**
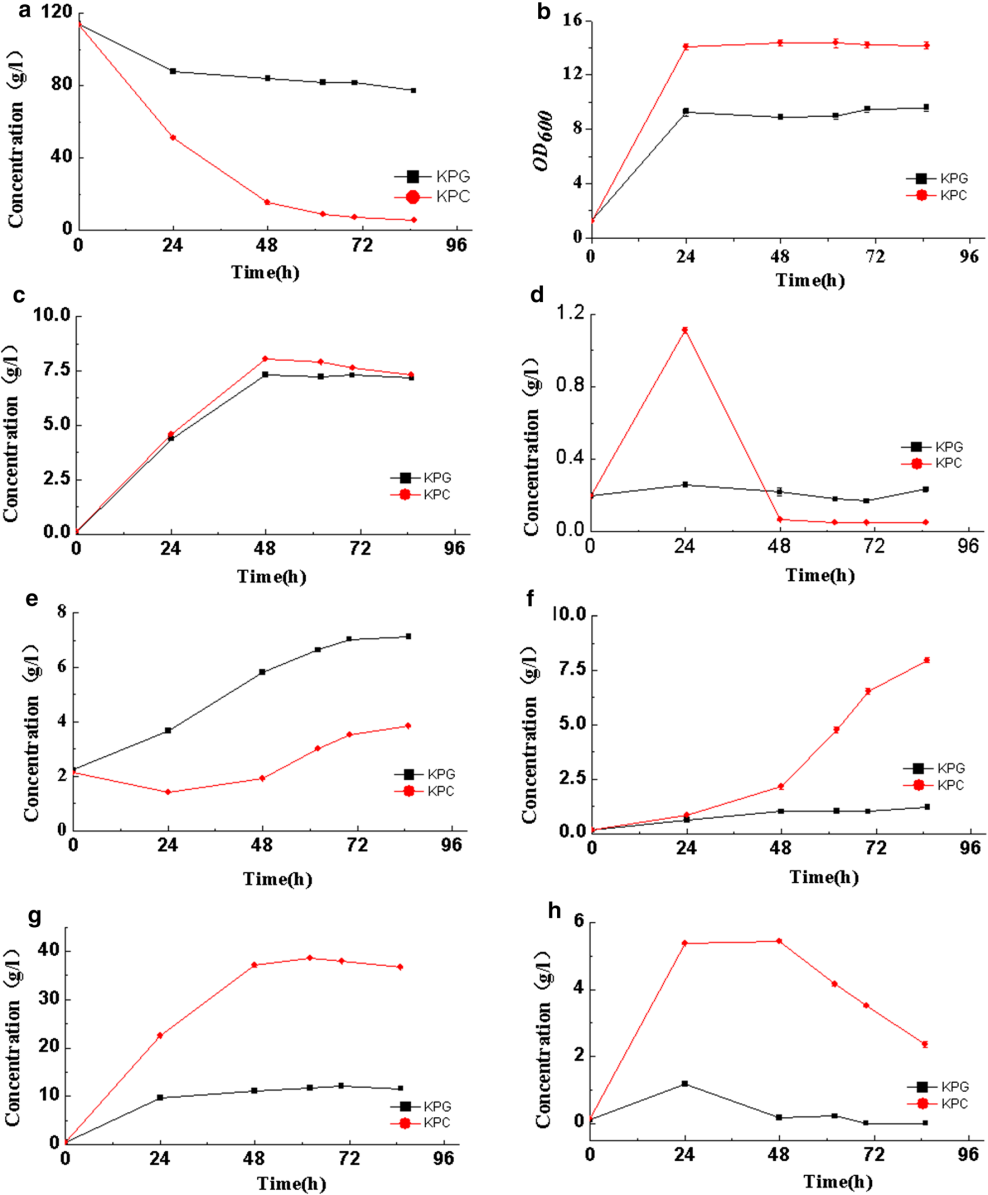
Results of fermentation with kpG (filled square) and kpC (filled circle) (**a** comparison of xylose consumptions, **b** growth curve, **c** succinic acid, **d** lactic acid, **e** acetic acid, **f** acetoin, **g** 2,3-butanediol, **h** ethanol)

*rpoD* is the primary coding gene for the most frequent transcription factor *σ*^70^ in *K. pneumoniae*, which allows the RNA polymerase core enzyme to recognize the promoter during transcription and greatly enhances the affinity of RNA polymerase to the DNA sequence of the promoter [18]. This gene can be used as the key element in gTME. Alper and co-workers reported the isolation of *E. coli* strain that is ethanol-tolerant with a doubling time of about 3.5 h in 50 g/L ethanol by mutating *rpoD* gene [21]. Herein, error-prone PCR was applied to introduce mutations to *rpoD* and a selected strain (kpC) with high tolerance toward xylose and high yield of 2,3-butanediol. In the batch fermentation, the consumption of xylose reached 107.7 g/L compared with the initial strain kpG, and the consumption of xylose increased by 2.89 times (Fig. 2a) after 85 h. In addition, the amount of biomass (OD600, Fig. 2b) also increased. The accumulation of acetoin and 2,3-butanediol increased by more than 7.36 and 3.25 times, respectively. Succinic acid had almost no change, and lactic acid was completely consumed. After a dynamic process, less than 4 g/L of lactic acid remained in the mutant, whereas that of the original strain reached 7.14 g/L. Other metabolites like lactic acid was accumulated in first 24 h but was consumed quickly later (Fig. 2c–h). Hence, compared with the original kpG strain, the selected mutant kpC could break the restriction of xylose and allow fermentation in a higher xylose concentration. We suspect that the xylose transport, signal transduction, and electron transfer abilities of the kpC strain may be increased by the mutation of the global transcriptional regulator *rpoD*. To further elucidate the molecular mechanism of high xylose tolerance, we performed transcriptomic analysis.

### Sequence alignment and mutational analysis of the mutant

**Fig. 3:**
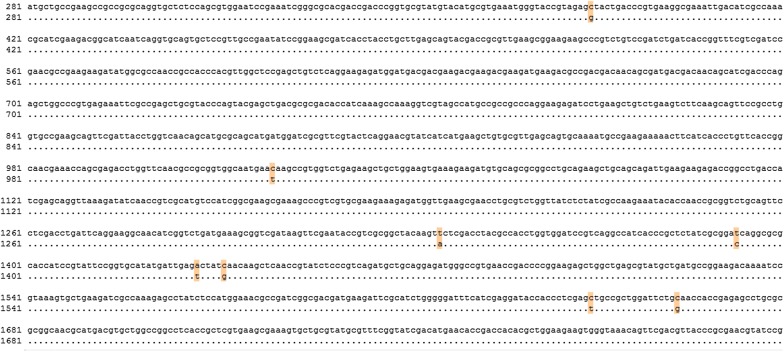
Summary of the eight mutation sites found in the gene sequence of RpoD in xylose-tolerant mutant KpC

**Fig. 4:**
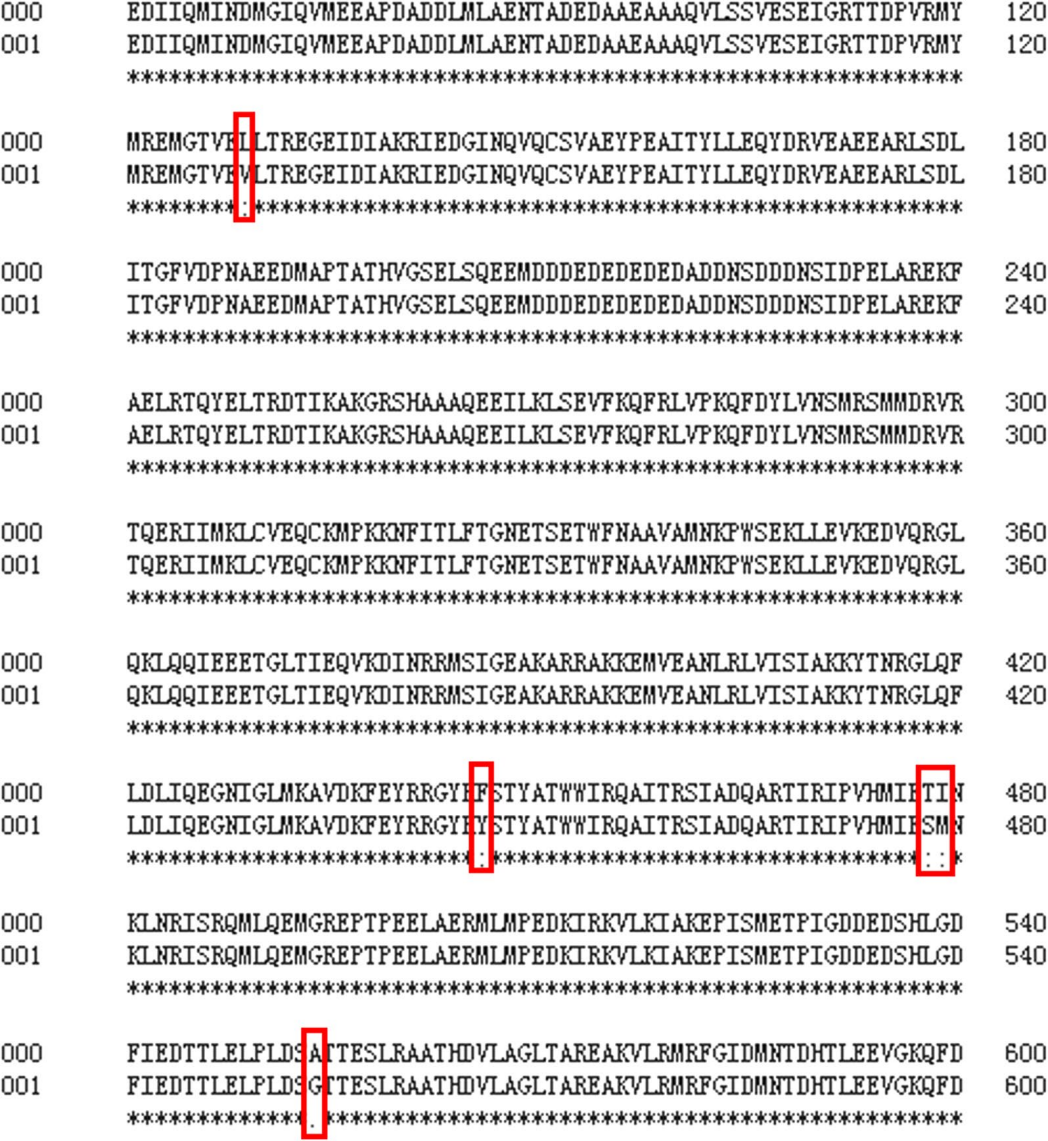
Summary of the five mutation sites found in the primary sequence of RpoD in xylose-tolerant mutant KpC

Mutant *rpoD* genes were sequenced using the primers RpoD-Sence-EcoR and RpoD-Anti-Xba I (Additional file 2: Table S4). The DNA and amino acid sequences were aligned and compared using Clone Manager Suite v7 and MEGA 6.0, respectively. The analysis of the mutations found in kpC revealed several interesting features. Figure 3 shows that eight total point mutations were introduced into the gene sequence of *rpoD* in the kpC strain of *K. pneumoniae* through error-prone PCR. These mutations include five non-synonymous mutations (L129V, T446Y, T478S, I479M, and A554G) and three same-sense mutations that provide the high xylose tolerance and high yield of 2,3-butanediol. Their amino acid substitutions are summarized in Fig. 4.

Tan et al. [22] found that *rpoD* is an RNA polymerase sigma subunit composed of an N-terminal domain of regions 1.1 (residues 18–88) and 1.2 (residues 116–149), a non-essential region (residues 245–405), and a C-terminal domain of regions 2 (residues 437–507), 3 (residues 516–593), and 4 (residues 599–657) (NCBI-conserved domain 2015). Among these mutations, three-point mutations (T446Y, T478S, and I479M) fell into the conserved region 2, and one substitution (L129V and A554G) was present in regions 1.1 and 3. Hence, these mutations exert differential effects on the promoter recognition and transcription initiation.

The analysis of the mutations found in the above-mentioned mutants revealed several interesting features. First, simple modification of the sigma factor *rpoD* enhanced strain tolerance toward xylose and 2,3-butanediol yield. Second, mutations occurred in the predicted conserved regions (1.2, 2, and 3), and none of the mutations were located at non-essential regions. Furthermore, the three same-sense mutations made non-function?? from the amino acid level, which may promote the gene translation by replacing the common codon for rare codons, thus preventing blockage of translation due to the shortage of tRNAs for the rare codons [23].

### Transcriptional expression profiling of *K. pneumoniae*

From the cDNA library, 13,158,224 and 12,603,262 raw reads were obtained through Illumina HiSeq 2000 sequencing, corresponding to the kpG and kpC’s samples, named kpG_48 and kpC_48, respectively. Q20 bases with base quality above 20 and error rate below 0.01% accounted for more than 97.5% of the total, thereby demonstrating that the raw sequencing reads were highly credible. After a stringent quality assessment and data filtration, 12,287,332 and 11,974,748 clean, high-quality reads containing, respectively, 1.14 and 1.12 Gb of 100% Q20 bases(Additional file 2: Table S1) were obtained for further analyses. Next-Generation Sequencing short-read sequences were then assembled into 9774 unigenes using the Trinity de novo assembly software. N50 refers to the length of the smallest transcripts in the set containing the fewest (largest) transcripts combined length of which represented at least 50% of the assembly. The length statistics of kpG and kpC were 1841 and 2265 bp, and the mean lengths of the unigenes were 894 bp and 976 bp, respectively. An overview of the assembly is shown in Additional file 2: Table S2. These unigenes represent all putative, non-overlapping protein-coding sequences expressed by *K. pneumoniae* cultured under the given conditions.

First, all unigenes were annotated based on BLASTX homology searches (*E*-value < 10^−5^) against the NCBI Nr and Swiss-Prot, as well as with the sequence-search facilities in-built with the Clusters of Orthologous Groups (COG), Gene Ontology (GO), and Kyoto Encyclopedia of Genes and Genomes (KEGG) databases. Afterward, the genes were compared against the nucleic acid database Nt based on BLASTN (*E*-value < 10^−5^). The results are listed in Additional file 2: Table S3.

**Fig. 5:**
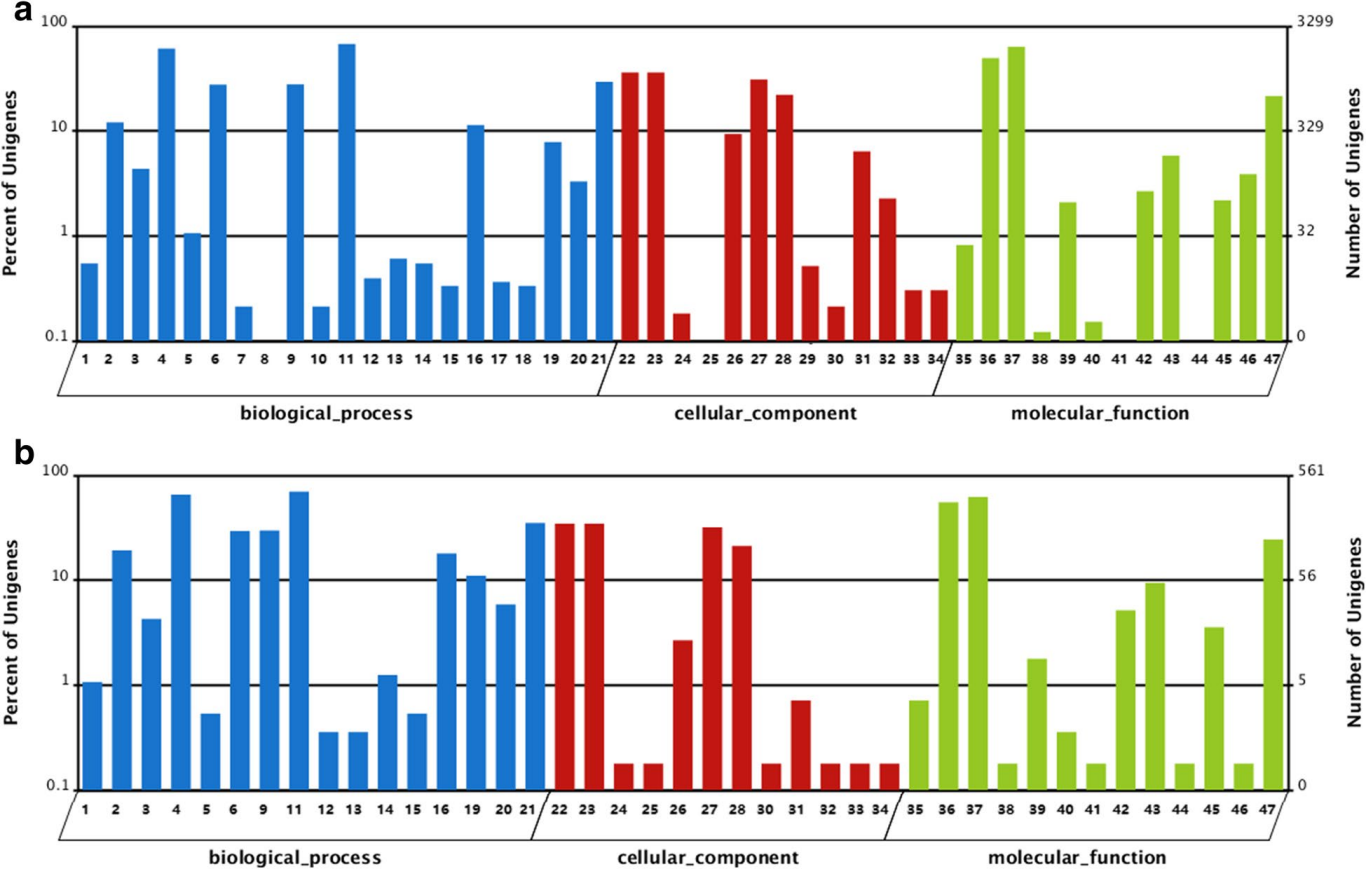
GO analysis results (**a** GO classification of all unigene, **b** GO analysis of DEGs). 1: biological adhesion; 2: biological regulation; 3: cellular component organization or biogenesis; 4: cellular process; 5: developmental process; 6: establishment of localization; 7: growth; 8: immune system process; 9: localization; 10: locomotion; 11: metabolic process; 12: multi-organism process; 13: multicellular organismal process; 14: negative regulation of biological process; 15: positive regulation of biological process; 16: regulation of biological process; 17: reproduction; 18: reproduction process; 19: response to stimulus; 20: signaling; 21: single-organism process; 22: cell; 23: cell part; 24: extracellular region; 25: extracellular region part; 26: macromolecular complex; 27: membrane; 28: membrane part; 29: membrane-enclosed lumen; 30: nucleoid; 31: organelle; 32: organelle part; 33: virion; 34: virion part; 35: antioxidant activity; 36: binding; 37: catalytic activity; 38: channel regulator activity; 39: electron carrier activity; 40: enzyme regulator activity; 41: metallochaperone activity; 42: molecular transducer activity; 43: nucleic acid binding transcription factor activity; 44: protein binding transcription factor activity; 45: receptor activity; 46: structural molecule activity; 47: transporter activity

Gene Ontology is a genes’ functional classification system, and it is used for annotating and analyzing gene functions and their products in various organisms. Its main categories include molecular functions, cellular components, and biological processes [24]. Blast2 GO was used to analyze the GO annotation of the assembled unigene [25], whereas WEGO was used for the visualization of the assigned GO functional classifications [26]. Figure 5a illustrates 3299 annotated unigenes (82.68% of all annotated unigenes) that were assigned with at least one GO term based on sequence similarity with known proteins annotated with GO terms. These unigenes were divided into three functional ontology categories: biological process, cellular component, and molecular function. Cell part and catalytic activity were the most abundant classes in the cellular component and molecular function categories, respectively. Metabolic process was the largest classification in the biological process, including the electron transport chain, cation transport, oxidation–reduction process, and transmembrane transport.

**Fig. 6:**
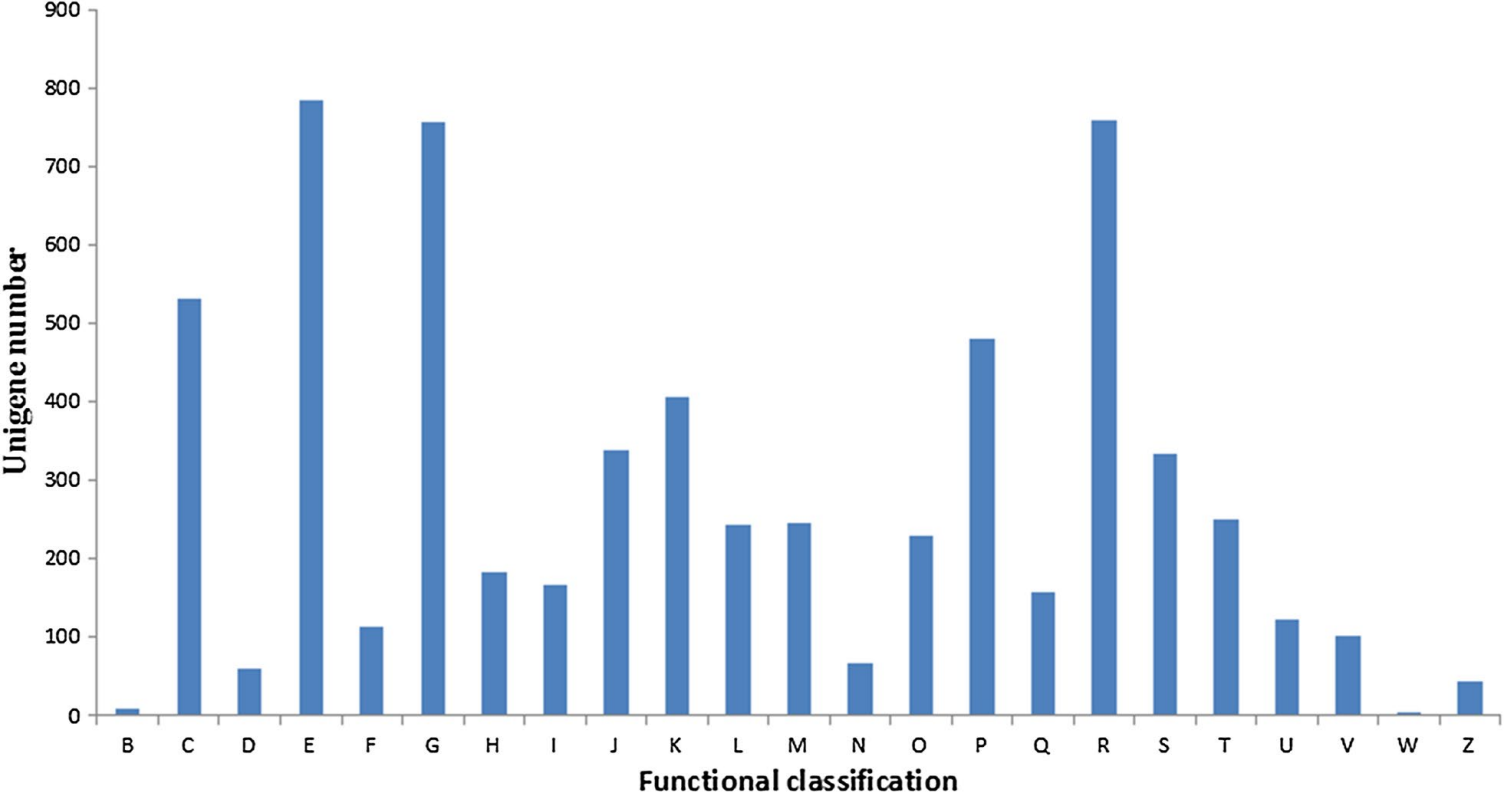
COG classification of all unigenes. The meaning of letters on the X-axis signifies as follows: chromatin structure and dynamics (B); energy production and conversion (C); cell cycle control, cell division, chromosome partitioning (D); amino acid transport and metabolism (E); nucleotide transport and metabolism (F); carbohydrate transport and metabolism (G); coenzyme transport and metabolism (H); lipid transport and metabolism (I); translation, ribosomal structure and biogenesis (J); transcription (K); replication, recombination, and repair (L); cell wall/membrane/envelope biogenesis (M); cell motility (N); posttranslational modification, protein turnover, chaperones (O); inorganic ion transport and metabolism (P); secondary metabolites biosynthesis, transport, and catabolism (Q); general function prediction only (R); function unknown (S); signal transduction mechanisms (T); intracellular trafficking, secretion, and vesicular transport (U); defense mechanisms (V); extracellular structures (W); and cytoskeleton (Z)

The COG database is used to classify the comprehensive complement of proteins encoded within a complete genome. Each COG refers to a group of orthologous proteins that are evolutionally conserved across different species. Thus, COG reflects the one-to-one, one-to-many, and many-to-many orthologous networks [27]. Functional annotation and classification were achieved by aligning the unigenes with COG entries, and showed that the 3309 unigenes could be classified into 23 categories (Fig. 6). The three largest categories were as follows: “Amino acid transport and metabolism (E),” “General function prediction only (R),” and “Carbohydrate transport and metabolism (G).” In addition, 532 unigenes were assigned to the “Energy production and conversion (C)” category, and 481 unigenes were assigned to the “Inorganic ion transport and metabolism (P)” category.

### Analysis of differential expression genes (DEGs)

FPKM calculates the expression quantity of all unigenes and locates DEGs with SOAP, where the false discovery rate is at the most 0.01 and multiple differences are more than twice. Compared with kpG, 500 unigene expression levels were upregulated, whereas 174 unigene expression levels were downregulated in kpC.

From the GO analysis, the DEGs were found in 41 categories, indicating that 87.23% of all categories were changed. Moreover, more than 50 unigene expression levels were regulated in 15 categories. Among them, the cell part and catalytic activity were the most abundant classes in the cellular component and molecular function categories, respectively. Metabolic process was the largest classification in the biological process (Fig. 5b).

**Fig. 7:**
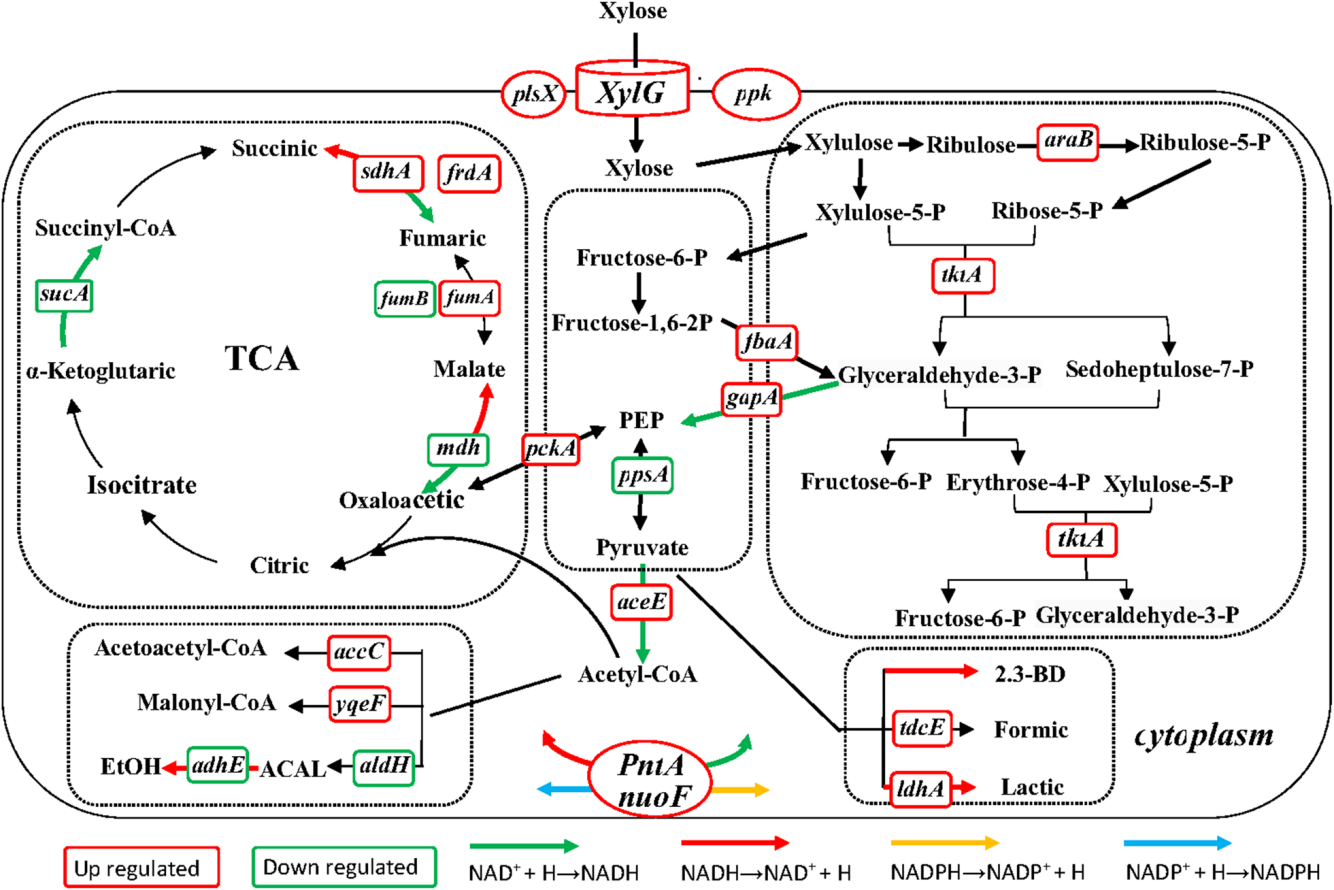
Xylose metabolism pathway of *K. pneumoniae*. Genes marked in *red* and *green* represent the upregulated and downregulated ones, respectively

**Fig. 8:**
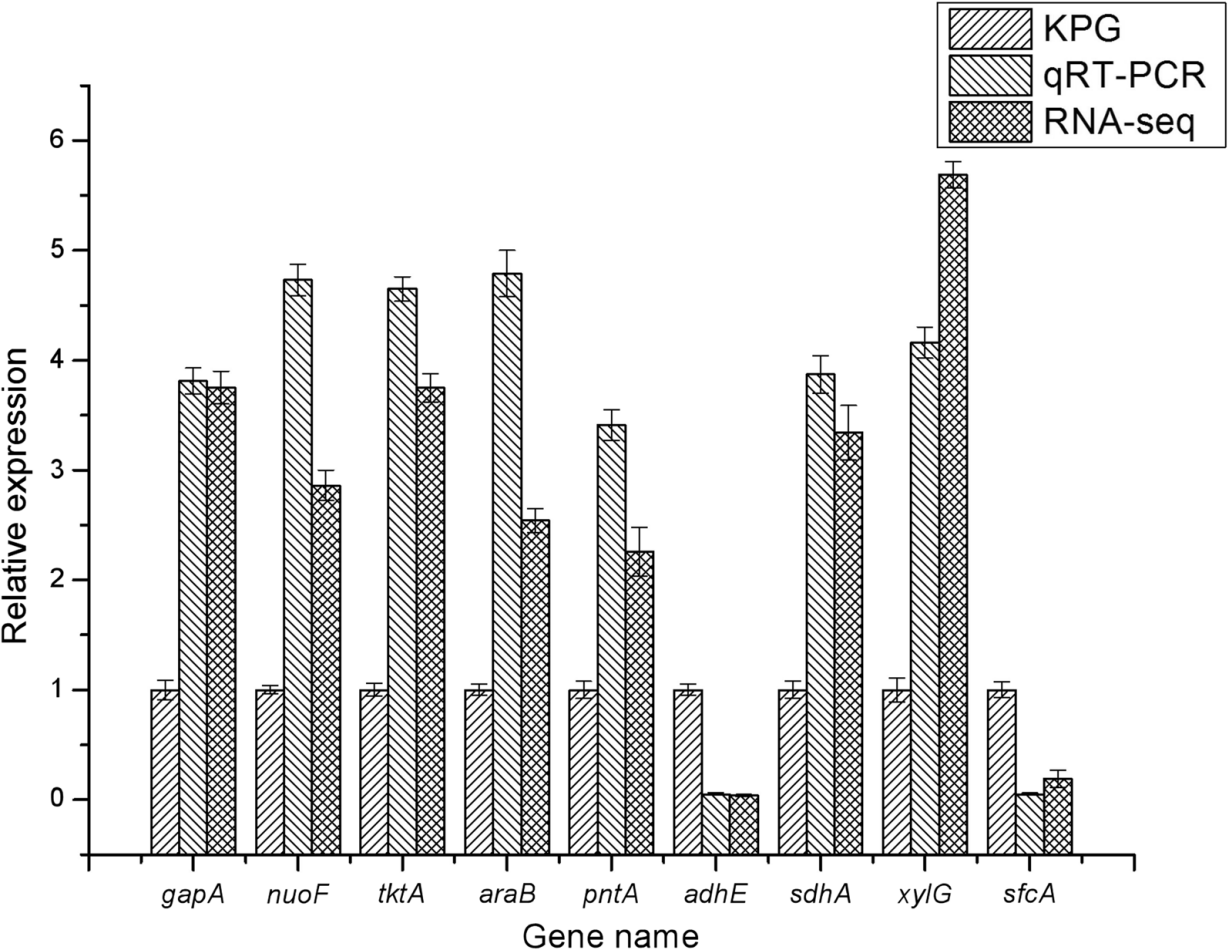
Unigene relative expression levels shown by qRT-PCR and RNA-seq

KEGG pathway was used to identify the biological pathways of the putative genes. The results suggested that 590 of the 674 DEGs were mapped into 114 pathways, 15 of which contain most of DEGs, as listed in Table 1. Table 2 shows 38 DEGs that are related to xylose metabolism and product synthesis, referring to multiple physiological and biochemical processes, such as glycolysis, TCA cycle, PPP, oxidative phosphorylation, energy metabolism, and transmembrane transport. According to the KEGG pathways, the metabolic network was constructed containing the xylose metabolic pathway, the formation of 2,3-butanediol and by-products, and those for related key enzymes (Fig. 7). To some extent, the ATP-binding proteins of the xylose transport system, the glycerol-3-phosphate acyltransferase, and the phosphate kinase in *K. pneumoniae*, encoded by *xylG*, *plsX*, and *ppk*, which affect the transfer of xylose from the extracellular matrix into the cells, were upregulated by 5.7-, 2.2-, and 3.0-folds, respectively. Compared with the parent strain, the key transcription of genes in the PPP was enhanced in the kpC strain. The mRNA fragments of *araB* and *tktA* increased by 2.5 and 2.7 times, respectively. The results show that the PPP of the kpC strain was higher than that of parental strain, allowing the mutant strain to produce more NADPH + H^+^, a hydrogen donor involved in various metabolic reactions [28]. The transcription of the coding genes, *fbaA* and *gapA*, which participate in glycolysis and other metabolic pathways and provide energy and substrate for the synthesis of biological substances, were upregulated by 2.2- and 3.8-fold, respectively. Consequently, it increased the consumption rate of xylose in the kpC strain. Pyruvate is at a pivotal junction of the metabolism, and it determines the carbon fluxes for the TCA cycle or the formation of 2,3-butanediol. The transcription of malate dehydrogenase encoded by *mdh* involved in the TCA cycle was decreased by 2.5 times. However, the growth rate and biomass of strain kpC were much larger than those of the wild-type strain. Therefore, malate dehydrogenase has other functions, which is supported by previous study [29]. The transcriptions of the coding genes, *frdA* and *sdhA*, which are critical in the TCA cycle, were increased by 8.2 and 3.3 times, respectively. At the same time, the transcription of phosphoenolpyruvate carboxykinase coded by *pckA* was improved by sevenfold, allowing a higher proportion of carbon sources to enter the TCA cycle and producing more NADH and ATP in the strain of kpC. This finding is consistent with the increased transcription of *aceE* (Fig. 8). Consistent with the improvement of the TCA pathway, the transcription of genes through electronic transmission and the reduction of coenzyme coded by *pntA* and *nuoF* were increased by 3.6- and 2.9-fold, respectively. The ability of the mutant strain kpC obtained by directional evolution is superior to that of the parent strain in terms of signal transduction and electron transport, providing better a fermentation performance and increased 2,3-butanediol production under high xylose pressure.

**Table 1:** Pathway analysis of differentially expressed genes (DEGs) in kpG_48-vs-kpC_48

Pathway	DEGs numbers	All-unigene numbers	*P* value	*Q* value	Pathway ID
Purine metabolism	23 (5.08%)	96 (24.73%)	0.00	0.31	ko00230
Lysine biosynthesis	8 (1.77%)	23 (34.78%)	0.01	0.31	ko00300
Lipopolysaccharide biosynthesis	7 (1.55%)	19 (36.84%)	0.01	0.31	ko00540
d-Alanine metabolism	2 (0.44%)	2 (100%)	0.02	0.46	ko00473
Flagellar assembly	5 (1.1%)	13 (38.46%)	0.02	0.46	ko02040
Pyrimidine metabolism	11 (2.43%)	46 (23.91%)	0.03	0.55	ko00240
Ubiquinone and other terpenoid-quinone biosynthesis	10 (0.95%)	12 (83.33%)	0.00	0.02	ko00130
Two-component system	78 (7.4%)	193 (40.41%)	0.00	0.05	ko02020
Pantothenate and CoA biosynthesis	16 (0.87%)	17 (94.12%)	0.00	0.03	ko00770
Fatty acid biosynthesis	10 (1.69%)	29 (34.48%)	0.02	0.86	ko00061
Biosynthesis of secondary metabolites	55 (17.86%)	463 (35.95%)	0.02	0.20	ko01110
Nicotinate and nicotinamide metabolism	5 (1.62%)	17 (29.41%)	0.01	0.20	ko00760
Phosphatidylinositol signaling system	6 (1.19%)	16 (37.5%)	0.02	0.26	ko04070
Oxidative phosphorylation	48 (9.52%)	104 (47.12%)	0.00	0.00	ko00190
Phosphotransferase system (PTS)	8 (3.96%)	68 (11.76%)	0.05	0.47	ko02060

**Table 2:** Analysis of differentially expressed genes with xylose metabolism and product synthesis

Gene ID	KO ID	Description in KEGG	FPKM		
			kpG_48	kpC_48	Ratio
CL59.Contig3_All	K00123	Formate dehydrogenase, alpha subunit	50.73	*143.07*	*2.82*
CL85.Contig1_All	K01623	Fructose-bisphosphate aldolase, class I	7.35	***1.74***	***0.24***
CL85.Contig2_All	K00875	d-Ribulokinase	15.05	*38.19*	*2.54*
Unigene1035_All	K00324	NAD(P) transhydrogenase subunit alpha	149.96	*338.43*	*2.26*
Unigene1076_All	K02112	F-type H+-transporting ATPase subunit beta	100.49	***23.72***	***0.24***
Unigene1083_All	K00163	Pyruvate dehydrogenase E1 component	113.86	*538.51*	*4.73*
Unigene1153_All	K01895	Acetyl-CoA synthetase	11.22	*31.42*	*2.80*
Unigene1155_All	K00540	[EC:1.-.-.-]	10.48	*22.74*	*2.17*
Unigene1156_All	K00134	Glyceraldehyde 3-phosphate dehydrogenase	32.59	***7.15***	***0.22***
Unigene120_All	K00016	l-Lactate dehydrogenase	9.72	*21.24*	*2.19*
Unigene1301_All	K00164	2-Oxoglutarate dehydrogenase E1 component	303.59	***135.99***	***0.45***
Unigene1302_All	K04072	Acetaldehyde dehydrogenase/alcohol dehydrogenase	259.56	***138.23***	***0.53***
Unigene1333_All	K00027	Malate dehydrogenase (oxaloacetate-decarboxylating)	423.22	***79.07***	***0.19***
Unigene1423_All	K01708	Galactarate dehydratase	3.32	*11.53*	*3.47*
Unigene1482_All	K02160	Acetyl-CoA carboxylase biotin carboxyl carrier protein	1.14	*26.21*	*22.99*
Unigene1622_All	K01610	Phosphoenolpyruvate carboxykinase (ATP)	45.82	*322.59*	*7.04*
Unigene1654_All	K01630	2-Dehydro-3-deoxyglucarate aldolase	8.13	*41.7*	*5.13*
Unigene1791_All	K00128	Aldehyde dehydrogenase (NAD+)	91.44	***56.81***	***0.62***
Unigene1824_All	K01679	Fumarate hydratase, class II	26.79	***8.34***	***0.31***
Unigene2070_All	K03621	Glycerol-3-phosphate acyltransferase PlsX	59.66	*129.23*	*2.17*
Unigene2154_All	K00344	NADPH2:quinone reductase	4.79	*9.76*	*2.04*
Unigene2315_All	K01198	Xylan 1,4-beta-xylosidase	44.25	*192.47*	*4.35*
Unigene2317_All	K01961	Acetyl-CoA carboxylase, biotin carboxylase subunit	29.16	*75.82*	*2.60*
Unigene2351_All	K00615	Transketolase	11.25	*30.88*	*2.74*
Unigene2391_All	K00134	Glyceraldehyde 3-phosphate dehydrogenase	3.81	*14.3*	*3.75*
Unigene24_All	K03885	NADH dehydrogenase	24.75	*70.73*	*2.86*
Unigene2609_All	K00244	Fumarate reductase flavoprotein subunit	1.89	*15.72*	*8.32*
Unigene29_All	K10545	d-Xylose transport system ATP-binding protein	70.37	*400.25*	*5.69*
Unigene35_All	K00322	NAD(P) transhydrogenase	39.52	*144.16*	*3.65*
Unigene531_All	K00029	Malate dehydrogenase (oxaloacetate-decarboxylating) (NADP+)	52.42	***21.1***	***0.40***
Unigene533_All	K00121	*S*-(hydroxymethyl)glutathione dehydrogenase/alcohol dehydrogenase	78.74	*187.57*	*2.38*
Unigene569_All	K01007	Pyruvate, water dikinase	109.29	***22.1***	***0.20***
Unigene65_All	K00937	Polyphosphate kinase	10.13	*25.14*	*2.48*
Unigene6_All	K00121	*S*-(hydroxymethyl)glutathione dehydrogenase/alcohol dehydrogenase	2057.89	***80.87***	***0.04***
Unigene792_All	K00239	Succinate dehydrogenase flavoprotein subunit	20.08	*67.09*	*3.34*
Unigene820_All	K01676	Fumarate hydratase, class I	7.64	*22.80*	*2.98*
Unigene835_All	K13953	Alcohol dehydrogenase, propanol-preferring	91.24	***27.82***	***0.30***
Unigene886_All	K00626	Acetyl-CoA *C*-acetyltransferase	5.87	*23.72*	*4.04*

The values given in italics and bold italics represent the upregulated and downregulated, respectively

### Confirmation of unigene expression through quantitative real-time quantitative reverse transcription PCR (qRT-PCR)

Nine unigenes were selected for qRT-PCR verification to confirm the accuracy and reproducibility of the results of the transcriptomic analysis by means of the cDNA of kpG and kpC as templates. The unigene primers are shown in Additional file 2: Table S4. The relative trend of unigene expression as indicated by qRT-PCR data was consistent with those derived from RNA-seq (Fig. 8), confirming the results of transcriptomic analysis.

### Confirmation of DEG function

Through gene overexpression and gene deletion, the function of the DEGs was confirmed. Seven gene overexpression strains and two gene deletion strains were obtained through the gene deletion in the synthesis of byproduct (*adhE*) and pyruvate metabolism (*sfcA*) and the gene overexpression in the transmembrane transport (*xylG* and *ppk*), PPP (*tktA* and *araB*), respiratory chain (*nuoF* and *pntA*), and glycolysis (*gapA*).

The consumption rate of xylose and the concentration of 2,3-butanediol obtained by means of these strains are shown in Table 3. The consumption rates of xylose were improved by 2.3% and 20.6%, and the production rates of 2,3-butanediol were improved by 9.8% and 23.7%, respectively, in the strains by overexpression of the xylose transporter ATP-binding subunit coding gene *xylG* and polyphosphate kinases coding gene *ppk* associated with transmembrane transport. Hence, the substrate uptake and product formation can be increased by ameliorating the transmembrane transport ability. Microbial tolerance usually varies with the transport ability of the transmembrane [30].

**Table 3:** Comparison of *K. pneumoniae* parent strain (kpG), overexpressing strains, and deleting strains in a 72-h flask cultivation; the values given represent mean ± SD

	Strains	Biomass (g/L)	Consumption of xylose (g/L)	2,3-BD concentration (g/L)	Utilization ratio of xylose (%)	2,3-BD yield (g/g)
	kpG	3.57 ± 0.05	45.49 ± 0.69	12.78 ± 0.13	37.9 ± 0.69	0.28 ± 0.01
Pentose phosphate pathway	kp-araB	3.72 ± 0.03	49.4 ± 0.87	14.22 ± 0.19	41.2 ± 0.72	0.29 ± 0.02
	kp-tktA	3.89 ± 0.03	55.22 ± 0.98	18.03 ± 0.15	46.0 ± 0.86	0.33 ± 0.05
Signal transduction and electronic transmission	kp-pntA	3.84 ± 0.02	50.72 ± 0.69	16.38 ± 0.22	42.3 ± 0.58	0.32 ± 0.02
	kp-nuoF	3.88 ± 0.07	54.77 ± 0.78	16.15 ± 0.12	45.6 ± 0.55	0.29 ± 0.04
Glycolysis	kp-gapA	3.91 ± 0.04	57.5 ± 1.01	14.34 ± 0.11	47.9 ± 0.72	0.25 ± 0.04
Transmembrane transport	kp-ppk	3.88 ± 0.05	54.86 ± 0.97	15.81 ± 0.07	45.7 ± 0.83	0.29 ± 0.01
	kp-xylG	3.69 ± 0.06	46.54 ± 0.99	13.03 ± 0.14	38.8 ± 0.56	0.3 ± 0.02
The synthesis of byproduct	ΔadhE	3.86 ± 0.03	52.27 ± 0.86	14.12 ± 0.12	43.6 ± 0.63	0.27 ± 0.03
	ΔsfcA	3.59 ± 0.03	45.58 ± 0.66	13.09 ± 0.11	40.0 ± 0.58	0.29 ± 0.03

By overexpressing the strains of *araB*, the coding gene of ribulose kinase, and *tktA*, the coding gene of transketolase, which are involved in the PPP, the consumption rates of xylose were improved by 8.6% and 21.4%, respectively, and the concentrations of 2,3-butanediol were increased by 11.3% and 41.1%, respectively. Hence, the improvement of the PPP could result in the production of a large quantity of NADPH by fermentation, providing the need for tissue metabolism [31].

By overexpressing *gapA*, the coding gene of glyceraldehydes-3-phosphate dehydrogenase, which plays a key role in the glycolytic pathway, the consumption rate of xylose was improved by 26.4%, and the concentration of 2,3-butanediol was increased by 12.2%. The glycolytic pathway is a common route for the catabolism of carbon in all organisms. It involves the degradation of carbon source to generate ATP, which provides energy for the growth and metabolism of microorganisms and provides intermediate products for the other metabolic pathways [32].

By overexpressing *pntA* and *nuoF*, the coding gene for NAD(P) transhydrogenase and NADH dehydrogenase that were related to NAD^+^/NADH and NADP^+^/NADPH recycling and electronic transmission, the consumption rate of xylose was improved by 11.5% and 20.4%, and the concentration of 2,3-butanediol was increased by 28.2% and 26.4%, respectively. Hence, the improved function of the respiratory chain enhanced the growth performance and the ability of the strain for production under high tolerance; moreover, it provides reference for the breeding of highly resistant strains [33].

By the deletion of *adhE*, the coding gene for alcohol dehydrogenase (the enzyme that regulates the synthesis of alcohol by-products), the consumption rate of xylose was improved by 14.9%, and the concentration of 2,3-butanediol was increased by 10.5%. Ethanol production pathway competes with the formation of 2,3-butanediol in NADH; hence, weakening the ethanol pathway increases the available NADH for the synthesis of 2,3-butanediol [20]. By deleting *sfcA*, the coding gene of malate dehydrogenase (oxaloacetate-decarboxylating) that catalyzes the formation of pyruvate form malate, the consumption rate of xylose, and the concentration of 2,3-butanediol were slightly increased. Hence, malic acid consumption and pyruvate generation can be evaluated by replacing the formation of pyruvate from malate, which will slightly affect the overall metabolism of the cells.

## Discussion

The direct or indirect manipulation of transcriptional regulatory networks has been a research focus to increase industrial-related microbial stress tolerance through engineered transcriptional regulatory proteins. Mounting numbers of regulators have been identified and characterized, and the directed evolution of global transcriptional regulators can improve the fermentation abilities of the strains [34]. Other techniques such as adaptive laboratory evolution, random mutagenesis, and rational engineering have been applied to increase the fermentation abilities of relative strains from xylose [35–37]. Adaptive evolution and mutagenesis are time consuming, and rational design is based on the current understanding of the pathway of product synthesis [38]. Global transcriptional regulators such as IrrE often activate multiple pathways, some of which may not be specific for targeting stress-tolerant phenotypes [39]. By combining gTME with transcriptomic analysis, new genes involved in the product synthesis could be determined. The enhancement of xylose tolerance in *K. pneumoniae* through directed evolution of global transcriptional regulators has not been reported. In this study, error-prone PCR was applied to introduce mutations to *rpoD* and a selected strain (kpC) with better tolerance toward xylose and higher yield of 2,3-butanediol. The sequence alignment of kpC and kpG is shown in the attached material, showing two mutations in the sites from C to T.

The *rpoD* gene is associated with the tolerance of different strains. Cyclohexane tolerance was improved by mutation of the global transcription factor *σ*^70^ (*rpoD*) of *E. coli*, and the results show that *E. coli* containing the *σ*^70^ mutant C9 can tolerate 69% of cyclohexane [40]. A three-step random insertion of deletional strand swap mutagenesis (RAISE) was applied to mutate the global transcriptional regulator *σ* (*rpoD*) to enhance the acid resistance of *E. coli* [41]. The mutant showed a much higher growth rate (0.22 h^−1^) than the parental strain (0.15 h^−1^) at a pH as low as 3.17. In this study, the kpC strain obtained by manipulating the *rpoD* gene consumed nearly 40 g/L of xylose, whereas the parent strain barely consumed 120 g/L of xylose after 24 h of fermentation (Fig. 2a). The biomass (OD600, Fig. 2b) is also increased compared with parent strain. Due the improved xylose tolerance, the kpC strain can consume more xylose, and the production of 2,3-butanediol production was 3.25 times higher than that of the parent strain. Moreover, by manipulating other global transcription factors, the tolerance of the respective strain can also be improved. Ethanol tolerance was improved by engineering the global transcription factor CRP of *E. coli* upon exposure to 150 g/L ethanol; the survival rate of the mutant strains after 15 min was about 12%, whereas that of the control strain was 0.01% [33]. The overexpression of the global transcriptional regulator IrrE in *E. coli* increased the tolerance to osmotic pressure, heat, and oxidative stress [39]. Still, the relationship between the global transcriptional regulators and the tolerance abilities requires further examination.

To identify the mechanism of the improved xylose tolerance in *K. pneumoniae* by manipulating the *rpoD* gene, transcriptome analysis was performed. From the RNA-seq data, the *σ* factor was able to simultaneously alter various metabolic functions, including carbon metabolism; TCA cycle; signal transduction; membrane transport; and carbohydrate, energy, nucleotide, and amino acid metabolism (Figs. 7, 8). At the same time, various structural and regulatory genes were upregulated or downregulated in response to xylose stressors and the metabolism in mutant kpC. Similar to our results, the transcriptomic analysis of CPR mutants with osmotic tolerance resulted in the upregulation or downregulation of 675 genes compared with the wild-type strain in response to osmotic tolerance [42]. The ATP-binding protein of the xylose transport system, the glycerol-3-phosphate acyltransferase, and the polyphosphate kinase in *K. pneumoniae* encoded by *xylG*, *plsX* and *ppk*, which affect the transfer of xylose from the extracellular matrix to the cells and the synthesis cell membrane, were upregulated by 5.7-, 2.2-, and 3.0-folds, respectively. The upregulation of these genes may alter the structure of the cell membrane and thereby increase the xylose tolerance of the strain [43–45]. This finding is consistent with the significant change in the genes involved in the synthesis of cell membranes and cell walls in *E. coli* under ethanol stress [46]. In contrast to the previous *E. coli* ethanol-tolerant strains, the expressions of central metabolic-related genes (*sdhA*, *frdA*, *fumA*, *tktA*, *araB*, *pckA*, *gapA*, *fbaA*, and *aceE*) were upregulated [46–49], allowing the mutant strain to produce more NADPH + H^+^, a hydrogen donor involved in various metabolic reactions [28]. However, ethanol-tolerant mutants screened by Chong et al. [33] have been reported on some intermediate metabolic (*aceE*, *acnA*, *sdhD*, *sucA*), ferrous ion transport (*entH*, *entD*, *fecA*, *fecB*), and general stress response (*osmY*, *rpoS*) genes in functional groups, which were upregulated.

By means *K. pneumoniae* kpG as the parental strain, the effects of the nine most promising genes on the consumption of xylose and the production of 2,3-butanediol were evaluated, where seven gene overexpression strains and two gene deletion strains were obtained. The fermentation performance of the respective mutant strains was evaluated at 120 g/L of xylose. The results of fermentation test revealed that four upregulated genes, namely *ppk*, *tktA*, *pntA*, and *nuoF*, which code polyphosphate kinase, transketolase, NAD(P) transhydrogenase, and NADH dehydrogenase, respectively, are essential for xylose consumption and 2,3-butanediol production. *ppk*, the coding gene for polyphosphate kinase, is associated with cell membrane composition and is a primary enzyme involved in polyp biosynthesis, which is crucial for cell growth and xylose consumption [50]. The yield of 2,3-butanediol was increased by 44%, and the consumption of xylose was increased by 21.4% by increasing the gene expression of *tktA*. The *tktA* gene is a key gene in the PPP, which plays an important role in the efficiency of xylose fermentation. Similarly, *Saccharomyces cerevisiae* was used to ferment xylose at an ethanol yield of 0.51 g/L/h by the overexpression of *tktA* (encoding transketolase) [51]. In addition, *tktA* is a major cold-resistant gene [52]. NAD(P) transhydrogenase catalyzes the chemical reaction NADPH + NAD^+^ → NADP^+^ + NADH, which could enhance the intracellular NADH/NAD^+^ ratio. The production of 2,3-butanediol requires the involvement of coenzyme NADH [53], which could be increased by the overexpression of *pntA*. Wang et al. [54] also increased the diol concentration, yield, and productivity to 110.8 g/L, 0.78 mol/mol, and 3.46 g/L/h, respectively, by increasing intracellular NADH/NAD^+^ ratio.

Membrane transhydrogenase, encoded by *pntA* operon, reduces NADP^+^ into NADPH and oxidizes NADH into NAD^+^, while re-importing protons [55]. The overexpression of *pntA* and *nuoF* associated with the increased respiratory chain function and export of protons and the overexpressions of *pntA* may indicate the need for higher promotive force to produce more NADPH from NADH. Besides, nuoF overexpression is consistent with the high ATP requirement for xylose import through the ABC transporter, XylFGH, supported by overexpression of *xylG* in the mutant strain. Also, the increased expressions of several genes involved in the PPP are consistent with the higher xylose import. Thus, assimilation is needed in the central metabolism. Thus, the increased consumption of xylose increases the fermentation performance.

## Conclusion

In conclusion, the modification of the *σ* factor at the gene level is an efficient method to improve the xylose tolerance of *K. pneumoniae*. Mutations were introduced into *rpoD* through error-prone PCR and an enrichment screening program to a variant (kpC) with enhanced xylose resistance. The results of transcriptomic analysis and functional gene validation show that several genes (*araB*, *tktA*, *pntA*, *gapA*, *nuoF*, *ppk*, and *xylG*) are essential in enhancing the xylose tolerance and the production of 2,3-butanediol by means of *K. pneumonia* that mainly involve glycolysis, TCA cycle, PPP, oxidative phosphorylation, energy metabolism, and transmembrane transport. Our study provides a new strategy for tolerance research and provides new information regarding the use of lignocellulosic biomass to produce fuel and chemicals economically.

## Materials and methods

### Strains and plasmids

The strains and plasmids with their relevant properties used in this study are shown in Table 4. The pUC18k plasmid containing the *lacZ* promoter and kanamycin resistance was used to overexpress the genes. The pMQ300 plasmid was used to clone the hygromycin-resistant gene. The pFLP2 plasmid was used to remove the hygromycin selection marker.

**Table 4:** Strains and plasmids used in this study

Strains or plasmids	Relevant properties	Reference or source
Strains		
*E. coli DH5α*	*supE44 ΔlacU169* (*ϕ 80 lacZΔM15*) *hsdR17 recAl endAl gyrA96 thi*-*1 relA*	TaKaRa
*E. coliS17*-*1λpir*	*TpR SmR recA thi*-*1 pro* hsdR-M^+^RP4:2-Tc:Mu: Km Tn7 *λ*pir	[50]
kpG	*Klebsiella pneumonia* CICC 10781	[51]
kpC	kpG directed evolution	This study
kp-araB	kpG containing plasmid pUC18k- araB	This study
kp-tktA	kpG containing plasmid pUC18k-tktA	This study
kp-pntA	kpG containing plasmid pUC18k-pntA	This study
kp-gapA	kpG containing plasmid pUC18k-gapA	This study
kp-nuoF	kpG containing plasmid pUC18k-nuoF	This study
kp-ppk	kpG containing plasmid pUC18k-ppk	This study
kp-xylG	kpG containing plasmid pUC18k-xylG	This study
ΔadhE	An alcohol dehydrogenase gene-deficient mutant of kpG	This study
ΔsfcA	An malate dehydrogenase gene-deficient mutant of kpG	This study
Plasmids		
pUC18k	Kam^r^, pUC ori, P_lac_, MCS	Lab archive
pDK7	Cm^r^	Lab archive
pUC18k-araB	Kam^r^, pUC18k containing araB gene	This study
pUC18k-xylG	Kam^r^, pUC18k containing xylG gene	This study
pUC18k-tktA	Kam^r^, pUC18k containing tktA gene	This study
pUC18k-pntA	Kam^r^, pUC18k containing pntA gene	This study
pUC18k-gapA	Kam^r^, pUC18k containing gapA gene	This study
pUC18k-nuoF	Kam^r^, pUC18k containing nuoF gene	This study
pUC18k-ppk	Kam^r^, pUC18k containing ppk gene	This study
pJTOOL-3	Suicide vector hph^r^ SacB oriT	Lab archive
pMQ300	hph^r^	Lab archive
pFLP2	Amp^r^ encodes FLP recombinase	Lab archive
p-adhE	Hph^r^, pJTOOL-3 derivative, where a adhE::HphFRT cassettes was inserted	This study
p-sfcA	Hph^r^, pJTOOL-3 derivative, where a sfcA::HphFRT cassettes was inserted	This study

### Disruption of the downregulated genes and overexpression of the upregulated genes

All primers used in this study are listed in Additional file 2: Table S4. For the disruption of the *adhE* and *sfcA*, the suicide vectors of pJTOOL-3-adhE and pJTOOL-3-sfcA were constructed for homologous recombination. The upstream and downstream homologous fragments of *adhE* and *sfcA* were amplified from the *K. pneumoniae* genome by means of primers adhE-US/R and adhE-DS/R and primers sfcA-US/R and sfcA-DS/R, respectively. The FRT-flanked hygromycin resistance (*hph*) was amplified using hphS and hphR. pJTOOL-3-adhE and pJTOOL-3-sfcA were constructed by inserting adhE::HphFRT (*adhE* upstream homologous fragment, FRT-flanked *hph* and *adhE* downstream homologous fragment connected) and sfcA::HphFRT (*sfcA* upstream homologous fragment, FRT-flanked *hph* and *sfcA* downstream homologous fragment connected) units into the *Xba*I restriction sites of pJTOOL-3, respectively. Then, conjugation and allelic exchange were performed, following the methods of Jon Jurriaan et al. [56]. To eliminate the hygromycin-resistant gene from the genome after deleting the target gene, the FLP expression plasmid was transformed into the cells. The PCR and digested products were purified using Omega PCR Purification Kit obtained from the USA Omega Bio-Tek (Guangzhou, China). DNA Assembly Master Mix kits were purchased from NEB (Beijing, China). The primers were synthesized by Jin Wei Zhi Biological Technology (Jiangsu, China).

The vector pUC18k was used for the overexpression of the genes in the genome of *K. pneumoniae*. The gene fragments were amplified through PCR by means of the genomic DNA of *K. pneumoniae* as templates with the S/R primers. Then, the fragments were ligated to the linearized pUC18k by means of NEB’s DNA Assembly Master Mix kit to generate the recombinant vectors. Finally, *K. pneumoniae* (kpG strain) electrocompetent cells were prepared and transformed using 10 ng of recombinant vectors following the standard methods [55]. Consequently, overexpressed strains were formed.

### Media and culture conditions

*Escherichia coli* DH5α was routinely cultured at 37 °C in a Luria–Bertani (LB) medium containing 1% peptone, 1% sodium chloride, and 0.5% yeast extract. For the selection of transformations, 50 mg/mL Cm was added to a final concentration of 0.12 mg/mL.

*Klebsiella pneumoniae* was cultured at 35 °C in LB medium. For the growth and fermentation, d-xylose was used as carbon source yielding 10.0 g/L yeast extract, 6.0 g/L KH_2_PO_4_, 14.0 g/L K_2_HPO_4_, 2.0 g/L (NH_4_)_2_SO_4_, 4.0 g/L sodium citrate, 4.0 g/L sodium acetate, and 0.4 g/L MgSO_4_·7H_2_O. When cultured with shake flask, the conditions were as follows: 35 °C, 150 rpm, and pH adjustment to 6.0 during medium preparation. At a total volume of 1 L and fluid volume of 0.7 L, the conditions are set as follows: 250 rpm of agitation, 0.4 L/min dissolve oxygen (DO), and pH 6.0. Throughout the whole process, the concentrations of xylose and metabolites such as succinic acid, lactic acid, acetic acid, acetoin, 2,3-butanediol, ethanol, and biomass (with OD_600_ value representation) were detected regularly through HPLC.

### Library construction and selection

In this work, the *rpoD* gene was subjected to random mutagenesis through error-prone PCR. An error-prone PCR reaction system was established by changing the proportions of four different deoxy-ribonucleoside triphosphates (dATP/dGTP/dCTP/dTTP = 1/1/5/5) and the concentrations of Mg^2+^ and Mn^2+^ (Additional file 2: Table S5). According to the differentiation of the concentrations of Mg^2+^ and Mn^2+^, the systems were named from A1 to C7 (Additional file 2: Table S6), respectively. Then, the optimal temperature of each system was tested, and the results are shown in Additional file 2: Table S7. After amplification (Additional file 1: Figure S1), the *rpoD* genes were purified and connected with plasmid pDK7, and then transformed to the competent cells of *E. coli* DH5α. Through resistant plate screening, plasmid extraction was conducted using a plasmid extraction kit (purchased from Solarbio), and verification was performed through PCR and agarose gel electrophoresis. Consequently, we acquired 91 different plasmids containing the mutant gene of *rpoD* and named them according to the system (Additional file 2: Table S8). These plasmids constitute the mutant plasmid library and were stored at − 20 °C.

The mutant strain library of *K. pneumoniae* was built on the basis of these plasmids through electroporation. Screening and verification were conducted through the same method as for *E. coli.* Lastly, 523 mutants were acquired. All mutants were tested through shake flask culture by means of xylose (120 g/L) as the carbon source. After 72 h, the biomass and concentration of xylose were detected, and the phenotypic excellent strains were selected. Finally, an excellent phenotype, kpC, was acquired.

### RNA extraction, cDNA library construction, and RNA-seq

For transcriptome sequencing, the cells were separated from the fermentation medium (the xylose concentration was 120 g/L) and cultured at 48 h. According to the manufacturer’s guidelines, mRNA selection, library preparation, and sequencing were performed using an Illumina HiSeq 2000 system after the extraction of total RNA by means of the RNA easy mini Kit (Qiagen, Germany). The quality and quantity of the mRNA samples were assessed using a nanophotometer (IMPLEN, USA), a qubit 2.0 fluorometer (Life Technologies), and an Agilent 2100 RNA Nano 6000 assay Kit (Agilent Technologies, USA). The cDNA library was constructed from the mRNA samples with high sample purity and integrity by means of random hexamers. After modification and enrichment prior to the attachment to the Illumina flow cell, the resulting cDNA library products were subjected to sequencing by means of the Illumina HiSeq 2000; the obtained data were translated into raw reads through base-calling [57].

### De novo transcriptome assembly and analysis

Low-quality reads were removed before the assembly and annotation assignment. Reads are considered with low quality if part of the reads contain adaptor, if part of reads has a ratio of uncertain bases of more than 5%, and if the quality score is less than 20 (a score of 20 corresponds to a 1% expected error rate). Ultimately, clean reads, which are available for de novo transcriptome assembly, were obtained. Considering the unavailability of the reference *K. pneumoniae* CICC 10781 genome, the Trinity software package was used to assemble its transcriptome by means of a k-mer cutoff value of 25 [60]. The redundancy of the obtained unigenes from the assembly were eliminated and then further assembled. After homologous transcript clustering with Phrap, a cluster was formed from several genes with high similarity of several genes [58, 59].

All unigenes were screened for functional annotation by means of BLAST against the protein sequence portion of several databases including non-redundant (Nr) proteins at NCBI, Swiss-Prot, the KEGG pathways, and COG by means of an expectation-value (*E*-value) cutoff 10^−5^ [60].

FPKM was used to calculate the expression quantity of all unigenes, showing DEGs with SOAP. The false discovery rate is at most 0.01, and multiple differences is more than twice. Acquired differential expression unigenes were mapped to the GO and KEGG pathways [60, 61].

### qRT-PCR

The mRNA of *K. pneumoniae* was extracted using the RNA kit (Omega, Madison, United States). PrimeScript reverse transcriptase (Takara, Japan) was used to synthesize the complementary DNA as the template of each gene. The DEGs were evaluated through qRT-qPCR by means of an Ultra SYBR Two-Step RT-PCR kit with ROX (reference dye for real-time PCR; CWBIO, China). The DNA recombination and repair protein gene (*recA*), which had a relatively stable expression level, were used as the reference genes. The primers for qRT-PCR are listed in Additional file 2: Table S4. The relative expression of transcription of the DEGs in the strains compared with the reference strain was analyzed using the ΔΔ*C*(*t*) method.

### Statistical analysis

The statistical significances of all the data were confirmed through standard ANOVA. A two-tailed test was used in this study. Differences with a *P* value < 0.05 were considered statistically significant. The experiments were performed in triplicates.

## Additional files

**Additional file 1: Figure S1.** Amplification product of 21 error-prone PCR systems (M: DL 5000 marker; 1–21: PCR product corresponding to each error-prone PCR system from A1 to C7 as in Additional file 2: Table S3).

**Additional file 2: Table S1.** Output statistics of de novo sequencing. **Table S2.** Statistics of unigene. **Table S3.** Statistics of annotated unigene. **Table S4.** Primers used in this study. **Table S5.** Error-prone PCR reaction systems. **Table S6.** 21 systems of error-prone PCR. **Table S7.** Annealing temperature of each error-prone PCR system (measured in degrees celsius). **Table S8.** Plasmid library.

## Abbreviations

ADP: adenosine diphosphate
ATP: adenosine triphosphate
DEGs: differential expression genes
DO: dissolve oxygen
*E. coli*: *E*sch*e*richia *coli*
gTME: global transcription machinery engineering
LB: Luria–Bertani Broth
NAD^+^: nicotinamide adenine dinucleotide phosphate
NADPH: nicotinamide adenine dinucleotide phosphate plus hydrogen
NGS: next generation sequencing
PCR: polymerase chain reaction
RNA-seq: RNA sequencing
XR: xylose reductase
XDH: xylitol dehydrogenase
XI: xylose isomerase

## References

[1] Jonker JGG, Hilst FVD, Junginger HM, Cavalett O, Chagas MF, Faaij APC (2015). Outlook for ethanol production costs in brazil up to 2030, for different biomass crops and industrial technologies. Appl Energy.

[2] Chen H, Qiu W (2010). Key technologies for bioethanol production from lignocellulose. Biotechnol Adv.

[3] Kötter P, Ciriacy M (1993). Xylose fermentation by *Saccharomyces cerevisiae*. Appl Microbiol Biotechnol.

[4] Kim JK, Seo SO, Yong CP, Yong SJ, Jin HS (2014). Production of 2,3-butanediol from xylose by engineered *Saccharomyces cerevisiae*. J Biotechnol.

[5] Matsushika A, Inoue H, Kodaki T, Sawayama S (2009). Ethanol production from xylose in engineered *Saccharomyces cerevisiae* strains: current state and perspectives. Appl Microbiol Biotechnol.

[6] Zhen C, Bo Z, Yin L (2012). Engineering *Saccharomyces cerevisiae* for efficient anaerobic xylose fermentation: reflections and perspectives. Biotechnol J.

[7] Jeffries TW (1983). Utilization of xylose by bacteria, yeasts, and fungi. Adv Biochem Eng Biotechnol.

[8] Khaliln AS, Collins JJ (2010). Synthetic biology: applications come of age. Nat Rev Genet.

[9] Zhang YP, Zhu Y, Zhu Y, Li Y (2009). The importance of engineering physiological functionality into microbes. Trend Biotechnol.

[10] Zhu LJ, Zhu Y, Zhang YP, Li Y (2012). Engineering the robustness of industrial microbes through synthetic biology. Trend Biotechnol.

[11] Alper H, Jin YS, Moxley JF, Stephanopoulos G (2005). Identifying gene targets for the metabolic engineering of lycopene biosynthesis in *Escherichia coli*. Metab Eng.

[12] Alper H, Miyaoku K, Stephanopoulos G (2005). Construction of lycopene-overproducing *E*. *coli* strains by combining systematic and combinatorial gene knockout targets. Nat Biotechnol..

[13] Gerber HP, Seipel K, Georgiev O, Höfferer M, Hug M, Rusconi S, Schaffner W (1994). Transcriptional activation modulated by homopolymeric glutamine and proline streches. Science.

[14] Kim JS, Kim J, Cepek KL, Sharp PA, Pabo CO (1994). Design of TATA box-binding protein/zinc finger fusions for targeted regulation of gene expression. Proc Natl Acad Sci USA.

[15] Park KS, Lee D, Lee H, Lee Y, Jang YS, Yong HK, Yang HY, Lee SI, Seol W, Kim JS (2003). Phenotypic alteration of eukaryotic cells using randomized libraries of artificial transcription factors. Nat Biotechnol.

[16] Alper H, Moxley J, Nevoigt E, Fink GR, Stephanopoulos G (2006). Engineering yeast transcription machinery for improved ethanol tolerance and production. Science.

[17] Zhang N, Buck M (2015). Perspective on the enhancer dependent bacterial RNA polymerase. Biomolecules.

[18] Burgess RR, Anthony L (2001). How sigma docks to RNA polymerase and what sigma dose. Curr Opin Microbiol.

[19] Paget MS (2012). Bacterial sigma factors and anti-sigma factors: structure, function and distribution. Biomolecules.

[20] Guo XW, Cao CH, Wang YZ, Li CQ, Wu MG, Chen YF, Zhang CY, Pei HD, Xiao DG (2014). Effect of the inactivation of lactate dehydrogenase, ethanol dehydrogenase, and phosphotransacetylase on 2,3-butanediol production in *Klebsiella pneumoniae* strain. Biotechnol Biofuels.

[21] Alper H, Stephanopoulos G (2007). Global transcription machinery engineering: a new approach for improving cellular phenotype. Metab Eng.

[22] Tan F, Wu B, Dai L, Qin H, Shui Z, Wang J, Zhu Q, Hu G, He M (2016). Using global transcription machinery engineering (gTME) to improve ethanol tolerance of *Zymomonas mobilis*. Microb Cell Fact.

[23] Li F, Li Y, Sun L, Li X, Yin C, An X, Chen X, Tian Y, Song H (2017). Engineering *Shewanella oneidensis* enables xylose-fed microbial fuel cell. Biotechnol Biofuels.

[24] Kanehisa M, Goto S, Hattori M, Aokikinoshita KF, Itoh M, Kawashima S, Katayama T, Araki M, Hirakawa M (2006). From genomics to chemical genomics: new developments in KEGG. Nucleic Acids Res.

[25] Conesa A, Götz S, García-Gómez JM, Terol J, Talón M, Robles M (2005). Blast2GO: a universal tool for annotation, visualization and analysis in functional genomics research. Bioinformatics.

[26] Ye J, Fang L, Zheng H, Zhang Y, Chen J, Zhang Z, Wang J, Li S, Li R, Bolund L, Wang J (2006). WEGO: a web tool for plotting GO annotations. Nucleic Acids Res.

[27] Liu T, Zhu S, Tang Q, Chen P, Yu Y, Tang S (2013). De novo assembly and characterization of transcriptome using Illumina paired-end sequencing and identification of *CesA* gene in ramie (*Boehmeria nivea* L. Gaud). BMC Genomics..

[28] Yang M, Zhang X (2017). Construction of pyruvate producing strain with intact pyruvate dehydrogenase and genome-wide transcription analysis. World J Microbiol Biotechnol.

[29] Miller SS, Driscoll BT, Gregerson RG, Gantt JS, Vance CP (1998). Malate dehydrogenase (MDH): molecular cloning and characterization of five different forms reveals a unique nodule-enhanced MDH. Plant J.

[30] Chen T, Wang J, Zeng L, Li R, Li J, Chen Y, Lin Z (2012). Significant rewiring of the transcriptome and proteome of an *Escherichia coli* strain harboring a tailored exogenous global regulator IrrE. PLoS ONE.

[31] Ma X, Wang L, Huang D, Li Y, Yang D, Li T, Li F, Sun L, Wei H, He K, Yu F, Zhao D, Hu L, Xing S, Liu Z, Li K, Guo J, Yang Z, Pan X, Li A, Shi Y, Wang J, Gao P, Zhang H (2017). Polo-like kinase 1 coordinates biosynthesis during cell cycle progression by directly activating pentose phosphate pathway. Nature.

[32] Koebmann BJ, Westerhoff HV, Snoep JL, Nilsson D, Jensen PR (2002). The glycolytic flux in *Escherichia coli* is controlled by the demand for ATP. J Bacteriol.

[33] Chong H, Huang L, Yeow J, Wang I, Zhang HF, Song H, Jiang RR (2013). Improving ethanol tolerance of *Escherichia coli* by rewiring its global regulator cAMP receptor protein (CRP). PLoS ONE.

[34] Yu HY, Gerstein M (2006). Genomic analysis of the hierarchical structure of regulatory networks. Proc Natl Acad Sci USA.

[35] Li H, Zhang G, Dang Y (2016). Adaptive laboratory evolution of *Klebsiella pneumoniae* for improving 2,3-butanediol production. Bioengineered.

[36] Watanabe T, Watanabe I, Yamamoto M, Ando A, Nakamura T (2011). A uv-induced mutant of *Pichia stipitis* with increased ethanol production from xylose and selection of a spontaneous mutant with increased ethanol tolerance. Bioresour Technol.

[37] Lu X, Fu X, Zong H, Zhuge B (2016). Overexpressions of *xylA* and *xylB* in *Klebsiella pneumoniae* leads to enhanced 1,3-propanediol production by cofermentation of glycerol and xylose. J Microbiol Biotechnol.

[38] Chen X, Cong G, Liang G, Hu G, Luo Q, Jia L (2018). Dceo biotechnology: tools to design, construct, evaluate, and optimize the metabolic pathway for biosynthesis of chemicals. Chem Rev.

[39] Pan J, Wang J, Zhou ZZ, Zhou HF, Yan YL, Zhang W, Lu W, Ping SZ, Dai QL, Yuan ML, Feng B, Hou XG, Zhang Y, Ma RQ, Liu TT, Feng L, Wang L, Chen M, Lin M (2009). IrrE, a global regulator of extreme radiation resistance in *Deinococcus radiodurans*, enhances salt tolerance in *Escherichia coli* and *Brassica napus*. PLoS ONE.

[40] Zhang F, Qian X, Si H, Xu G, Han R, Ni Y (2015). Significantly improved solvent tolerance of *Escherichia coli*, by global transcription machinery engineering. Microb Cell Fact.

[41] Gao X, Jiang L, Zhu L, Xu Q, Xu X, Huang H (2016). Tailoring of global transcription sigma D factor by random mutagenesis to improve *Escherichia coli* tolerance towards low-pHs. J Biotechnol.

[42] Zhang HF, Chong HQ, Ching CB, Jiang RR (2012). Random mutagenesis of global transcription factor cAMP receptor protein for improved osmotolerance. Biotechnol Bioeng.

[43] Erbeznik M, Strobel HJ, Dawson KA, Jones CR (1998). The d-xylose-binding protein, XylF, from *Thermoanaerobacter ethanolicus* 39E: cloning, molecular analysis, and expression, of the structural gene. J Bacteriol.

[44] Gao Q, Shang Y, Huang W, Wang C (2013). Glycerol-3-phosphate acyltransferase contributes to triacylglycerol biosynthesis, lipid droplet formation, and host invasion in *Metarhizium robertsii*. Appl Environ Microbiol.

[45] Rashid MH, Rumbaugh K, Passador L, Davies DG, Hamood AN, Iglewski BH (2000). Polyphosphate kinase is essential for biofilm development, quorum sensing, and virulence of *Pseudomonas aeruginosa*. Proc Natl Acad Sci USA.

[46] Chen TJ, Wang JQ, Zeng LL, Li R, Li J, Chen Y (2012). Significant rewiring of the transcriptome and proteome of an *Escherichia coli* strain harboring a tailored exogenous global regulator IrrE. PLoS ONE.

[47] Horinouchi T, Tamaoka K, Furusawa C, Ono N, Suzuki S, Hirasawa T, Yomo T, Shimizu H (2010). Transcriptome analysis of parallel-evolved *Escherichia coli* strains under ethanol stress. BMC Genomics.

[48] Goodarzi H, Bennett BD, Amini S, Reaves ML, Hottes AK, Rabinowitz JD, Tavazoie S (2010). Regulatory and metabolic rewiring during laboratory evolution of ethanol tolerance in *E. coli*. Mol Syst Biol..

[49] Gonzalez R, Tao H, Purvis JE, York SW, Shanmugam KT, Ingram LO (2003). Gene array-based identification of changes that contribute to ethanol tolerance in ethanologenic *Escherichia coli*: comparison of KO11 (parent) to LY01 (resistant mutant). Biotechnol Prog.

[50] Shi T, Ge Y, Zhao N, Hu X, Yuan Z (2015). Polyphosphate kinase of *Lysinibacillus sphaericus* and its effects on accumulation of polyphosphate and bacterial growth. Microbiol Res.

[51] Kobayashi Y, Sahara T, Suzuki T, Kamachi S, Matsushika A, Hoshino T, Ohgiya S, Kamagata Y, Fujimori KE (2017). Genetic improvement of xylose metabolism by enhancing the expression of pentose phosphate pathway genes in *Saccharomyces cerevisiae*, IR-2 for high-temperature ethanol production. J Ind Microbiol Biotechnol.

[52] Tang B, Liu XJ, Shi ZK, Chen QD, Xu YX, Wang S, Zhang F, Wang SG (2017). Transcriptome analysis and identification of induced genes in the response of *Harmonia axyridis* to cold hardiness. Comp Biochem Physiol Part D Genomics Proteomics.

[53] Kim SJ, Sim HJ, Kim JW, Lee YG, Park YC, Seo JH (2017). Enhanced production of 2,3-butanediol from xylose by combinatorial engineering of xylose metabolic pathway and cofactor regeneration in pyruvate decarboxylase-deficient *Saccharomyces cerevisiae*. Bioresour Technol.

[54] Wang Meng, Zhou Yiou, Tan Tianwei (2017). Cofactor Engineering for Enhanced Production of Diols by Klebsiella pneumoniae From Co-Substrate. Biotechnology Journal.

[55] Yang TW, Rao ZM, Hu GY, Zhang X, Liu M, Dai Y, Xu MJ, Xu ZH, Yang ST (2015). Metabolic engineering of *Bacillus subtilis* for redistributing the carbon flux to 2,3-butanediol by manipulating NADH levels. Biotechnol Biofuels.

[56] van Aartsen JJ, Rajakumar K (2011). An optimized method for suicide vector-based allelic exchange in *Klebsiella pneumoniae*. J Microbiol Methods.

[57] Liu FL, Sun XT, Wang W, Liang Z, Wang F (2014). De novo transcriptome analysis-gained insights into physiological and metabolic characteristics of *Sargassum thunbergii* (Fucales, Phaeophyceae). J Appl Phycol.

[58] Grabherr MG, Haas BJ, Yassour M, Levin JZ, Thompson DA, Amit I, Adiconis X, Fan L, Raychowdhury R, Zeng Q, Chen ZH, Mauceli E, Hacohen N, Gnirke A, Rhind N, Palma F, Birren BW, Nusbaum C, Lindblad-Toh K, Friedmanet N (2011). Full-length transcriptome assembly from RNA-Seq data without a reference genome. Nat Biotechnol..

[59] Iseli C, Jongeneel CV, Bucher P (1999). ESTScan: a program for detecting, evaluating, and reconstructing potential coding regions in EST sequences. Proc Int Conf Intell Syst Mol Biol.

[60] Mortazavi A, Williams BA, Mccue K, Schaeffer L, Wold B (2008). Mapping and quantifying mammalian transcriptomes by RNA-Seq. Nat Methods.

[61] Audic S, Claverie JM (1997). The significance of digital gene expression profiles. Genome Res.

