# Supplementary figures and images for: Improved xylose tolerance and 2,3-butanediol production of Klebsiella pneumoniae by directed evolution of rpoD and the mechanisms revealed by transcriptomics
Source: Biotechnol Biofuels. 2018 Nov 9;11:307. doi: 10.1186/s13068-018-1312-8 (PMC6225576; doi:10.1186/s13068-018-1312-8)

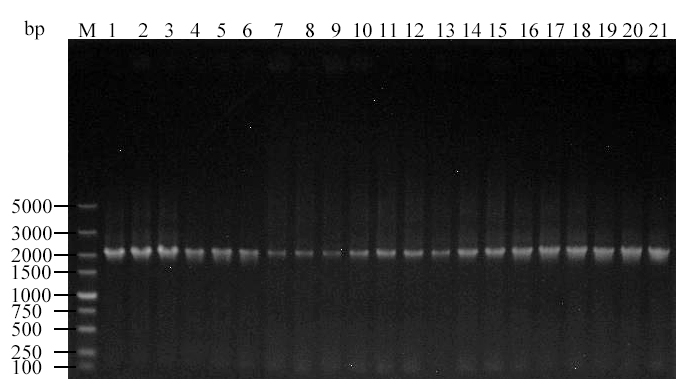

Supplement: Supplementary file 1 — Additional file 1: Figure S1. Amplification product of 21 error-prone PCR systems (M: DL 5000 marker; 1–21: PCR product corresponding to each error-prone PCR system from A1 to C7 as in Additional file 2: Table S3). [file 13068_2018_1312_MOESM1_ESM.tif]
