# Supplementary material for: Improved xylose tolerance and 2,3-butanediol production of Klebsiella pneumoniae by directed evolution of rpoD and the mechanisms revealed by transcriptomics
Source: Biotechnol Biofuels. 2018 Nov 9;11:307. doi: 10.1186/s13068-018-1312-8 (PMC6225576; doi:10.1186/s13068-018-1312-8)
Supplement: Supplementary file 2 — Additional file 2: Table S1. Output statistics of de novo sequencing. Table S2. Statistics of unigene. Table S3. Statistics of annotated unigene. Table S4. Primers used in this study. Table S5. Error-prone PCR reaction systems. Table S6. 21 systems of error-prone PCR. Table S7. Annealing temperature of each error-prone PCR system (measured in degrees celsius). Table S8. Plasmid library. [file 13068_2018_1312_MOESM2_ESM.doc]

Additional file 2

Table S1 output statistics of *de novo* sequencing

| Samples | Total Clean Reads | Total Clean Nucleotides (nt) | N percentage^*^ | GC percentage |
| --- | --- | --- | --- | --- |
| kpG_48 | 11974748 | 1197474800 | 0.01% | 53.43% |
| kpC_48 | 12287332 | 1228733200 | 0.01% | 56.25% |

* “N percentage” on behalf of the filrered uncertainty in proportion to the base.

| Sample | Total Number | Total Length(nt) | Mean Length(nt) | Distinct Clusters | Distinct Singletons |
| --- | --- | --- | --- | --- | --- |
| kpC_48 | 5117 | 4576907 | 894 | 177 | 4940 |
| kpG_48 | 4657 | 4545910 | 976 | 154 | 4503 |

Table S2 Statistics of Unigene

Table S3 Statistics of annotated Unigene

| Nr | Nt | Swiss-Prot | KEGG | COG | GO | All |
| --- | --- | --- | --- | --- | --- | --- |
| 3920 | 3496 | 3283 | 3414 | 3309 | 3299 | 3990 |

Table S4 Primers used in this study

| Primer name | Sequence (5′–3′) |
| --- | --- |
| RpoD-Sence-EcoR | CCGGAATTCTAAATGACAGACAATATCCTTC |
| RpoD-Anti-Xba I | TATTCTAGAAGACGATGCCCCGCGACGATTA |
| adhE-US | CCACCGCGGTGGCGGCCGCTCTCTGTGGGACTTTGACCTGC |
| adhE-UR | ATTCTCTAGAAAGTATAGGAACTTCTGGAGCAGATTAACGAGGCCT |
| adhE-DS | CTTTCTAGAGAATAGGAACTTCGAAGACACCTTCCGGATCGT |
| adhE-DR | GCCCGGGGGATCCACTAGTTCTAGACTGGTAGTAGTAGCCTATGCG |
| sfcA-US | CCACCGCGGTGGCGGCCGCTCTAGAACCGGTGAGATGAAGTGGTG |
| sfcA-UR | CTCTAGAAAGTATAGGAACTTCACAGCGACAGTTTACCGATC |
| sfcA -DS | ATACTTTCTAGAGAATAGGAACTTCGCGTGAAAGGCAAGCAGTATC |
| sfcA -DR | GCCCGGGGGATCCACTAGTTCTAGAGTTTCTGTCGATAAGCCAGCG |
| hphS | GAAGTTCCTATACTTTCTAGAGAATAGGAACTTCTCAGGCGCCGGGGGCGGTGT |
| hphR | TCCTATTCTCTAGAAAGTATAGGAACTTTAGAGCTTCAATTTAATTA |
| araB-S | CCATGGGCCCCTAGGAGATCTAATTGGGTTGGGATAATGC |
| araB-R | GTACGGACGTCCAGCTGAGATCTGGCACGCAAAGTCCGATCT |
| xylG-S | TGCTCTAGACTGTAACGTATCCGCAACCTC |
| xylG-R | TCCCCCGGGAAGTCGGTGGTAGTCCAG |
| tktA-S | CCATGGGCCCCTAGGAGATCTCCGACGCGCGAAATCATGAATACCGATCC |
| tktA-R | GTACGGACGTCCAGCTGAGATCTTGAGCTGGCAGGTTCGCGATGAC |
| pntA-S | CCATGGGCCCCTAGGAGATCTGATGGAAGGGAAAACTATGCGT |
| pntA-R | GTACGGACGTCCAGCTGAGATCTTTATCCTTTGCGAAACAT |
| gapA-S | CCATGGGCCCCTAGGAGATCTTTAGCGGGCAAATTTCTCC |
| gapA-R | GTACGGACGTCCAGCTGAGATCTATGAGTAAACTTGGGATTAATGG |
| nuoF-S | CCATGGGCCCCTAGGAGATCTACGCAAGGTCCGCAAAAGTT |
| nuoF-R | GTACGGACGTCCAGCTGAGATCTATGACCCCCGAACACCTTC |
| ppk-S | CCATGGGCCCCTAGGAGATCTAAGTATAACGCCGGGCAGAAG |
| ppk-R | GTACGGACGTCCAGCTGAGATCTGAATTATCCATGCAAAGTG |
| RT-gapA-S | GGGATCACCAGGCCAATG |
| RT-gapA-R | CCTATACCGGCACCCAGTC |
| RT-nuoF-S | AACGGATAGAAGGTGAGGGACT |
| RT- nuoF-R | GGTCTGTGGCTCGGGAAG |
| RT-tktA-S | GCCCGATTCATTGTAGGTCT |
| RT- tktA-R | GGCAGCGAGAAGAACGAG |
| RT-araB-S | ATAGATGTGCGGGCGATTC |
| RT- araB-R | TGGTGATTGGCGATAACGA |
| RT-pntA-S | CCAGCTCTACGGCACCAAC |
| RT- pntA -R | GCTGAGCGGAGACCTGAAT |
| RT-adhE-S | CGGGATTGTCGTGGAAGTA |
| RT-adhE-R | CGCCTGACAGAGGTTGGTT |
| RT-sdhA-S | CGTCTGCCGGGTATCCTT |
| RT-sdhA-R | GTCTTCGCCCTTCTCATTCA |
| RT-xylG-S | GGCGGCAACCAGCAAA |
| RT-xylG-R | CTCGGGCAGTTCGGATGA |
| RT-sfcA-S | TGCGTAACATCCAGGACACC |
| RT-sfcA-R | GGCGATTCTGGTAGGAGATAAAG |

Table S5 Error-prone PCR reaction systems

| Substrates | Contents（µL） |
| --- | --- |
| 10🞨PCR Buffer | 5 |
| dATP（100 mmol/L） | 0.1 |
| dGTP（100 mmol/L） | 0.1 |
| dCTP（100 mmol/L） | 0.5 |
| dTTP（100 mmol/L） | 0.5 |
| the upstream primer | 1 |
| the downstream primer | 1 |
| template | 1 |
| rTaq | 1 |
| Mn^2+^(10 mmol/L) | 0.5\1.5\2.5 |
| Mg^2+^(25 mmol/L) | 14\12\10\8\6\4\3 |
| ddH_2_O | up to 50 |

Table S6 21 systems of error-prone PCR

| Mg^2+^ (mmol/L)  Mn^2+^( mmol/L) | 7 | 6 | 5 | 4 | 3 | 2 | 1.5 |
| --- | --- | --- | --- | --- | --- | --- | --- |
| 0.1 | A1 | A2 | A3 | A4 | A5 | A6 | A7 |
| 0.3 | B1 | B2 | B3 | B4 | B5 | B6 | B7 |
| 0.5 | C1 | C2 | C3 | C4 | C5 | C6 | C7 |

Table S7 Annealing temperature of each error-prone PCR system（Measured in degrees Celsius）

|  | 1 | 2 | 3 | 4 | 5 | 6 | 7 |
| --- | --- | --- | --- | --- | --- | --- | --- |
| A | 64.0 | 64.0 | 64.0 | 64.0 | 58.4 | 58.4 | 55.6 |
| B | 64.0 | 64.0 | 64.0 | 58.4 | 60.0 | 55.6 | 55.6 |
| C | 62.0 | 62.0 | 62.0 | 62.0 | 62.0 | 57.0 | 55.6 |

Table S8 Plasmid library

| Systems | Plasmids |
| --- | --- |
| A1 | A1-1、A1-2、A1-3、A1-4、A1-5 |
| A2 | A2-1、A2-2、A2-3、A2-4 |
| A3 | A3-1、A3-2、A3-3、A3-4、A3-5 |
| A4 | A4-1、A4-2、A4-3、A4-4、A4-5 |
| A5 | A5-1、A5-2、A5-3 |
| A6 | A6-1、A6-2、A6-3、A6-4、A6-5 |
| A7 | A7-1、A7-2 |
| B1 | B1-1、B1-2、B1-3、B1-4、B1-5 |
| B2 | B2-1、B2-2、B2-3 |
| B3 | B3-1 |
| B4 | B4-1、B4-2、B4-3、B4-4、B4-5 |
| B5 | B5-1、B5-2、B5-3、B5-4、B5-5 |
| B6 | B6-1、B6-2、B6-3、B6-4、B6-5 |
| B7 | B7-1、B7-2、B7-3、B7-4、B7-5 |
| C1 | C1-1、C1-2、C1-3、C1-4 |
| C2 | C2-1、C2-2 |
| C3 | C3-1、C3-2、C3-3、C3-4、C3-5、C3-6、C3-7 |
| C4 | C4-1、C4-2、C4-3、C4-4、C4-5 |
| C5 | C5-1、C5-2、C5-3、C5-4、C5-5 |
| C6 | C6-1、C6-2、C6-3、C6-4、C6-5 |
| C7 | C7-1、C7-2、C7-3、C7-4、C7-5 |
